# Assessment of growth hormone gene polymorphism effects on reproductive traits in Holstein dairy cattle in Tunisia

**DOI:** 10.5194/aab-61-481-2018

**Published:** 2018-12-19

**Authors:** Sihem Amiri, Bayrem Jemmali, Mohamed Amine Ferchichi, Hajer Jeljeli, Rekik Boulbaba, Abderrahmane Ben Gara

**Affiliations:** 1 National Agronomic Institute of Tunisia, 43 Charles Nicoles street 1082, Tunis-Mahrajène, Tunisia; 2 Laboratory of Improvement and Integrated Development of Animal Productivity and Food Resources, Higher School of Agriculture of Mateur, University of Carthage, Tunis, Tunisia

## Abstract

Research to assess the effect of single genes on reproductive traits in bovine species is imperative to elucidate genes' functions and acquire a better perspective of quantitative traits. The present study was undertaken to characterize genetic diversity in the bovine growth hormone (GH) gene in a population of 410 Holstein dairy cows in Tunisia. The analyses were based on single nucleotide polymorphisms, and GH-*Alu*I and GH-*Msp*I detections and genotyping were carried out using the polymerase chain reaction-restriction fragment length polymorphism (PCR-RFLP) method. Data were analyzed using a mixed linear model with the MIXED procedure to reveal the possible effect of GH genotypes on reproductive traits. The frequency data of *Alu*I(L//V) and *Msp*I(+//-) alleles were 87.04//12.96 and 70.06//29.94, respectively. The distribution of the frequency of GH genotypes for LL/LV/VV and (-/-)//(+/-)//(+/+) were 77.75//18.59//3.66 and 15.37//29.13//55.50, respectively. The results of the statistical analyses proved that GH-*Alu*I showed a substantial favorable effect on exanimate traits except for the age at first calving; however, only a suggestive effect of GH-*Msp*I on the calving interval (CI) and the days open (DI) was found. The homozygous LL genotype seemed to be advantageous with respect to the CI and the DI compared with LV and VV genotypes. Heterozygous *Msp*I(+/-) cows tended to have a longer CI and DI than *Msp*I(+/+) and *Msp*I(-/-) cows, but the difference was not statistically significant. A significant effect of different GH-*Alu*I–*Msp*I combined genotypes was found on the number of inseminations per conception, the CI and the DI, and the LL/– combined genotype seemed to be associated with better reproductive performance. Based on these results, the LL genotype of the GH locus can be considered to be a favorable genotype for reproductive traits in Holstein dairy cattle, although these findings need to be confirmed by further research before polymorphisms can be used in a marker-assisted selection program.

## 1 Introduction

The availability of dense genomic information has increased genome-wide association research for quantitative traits in bovine species. Fertility is considered to be a functional polygenic trait controlled by numerous genetic loci and biased by environmental factors. Hence, linked candidate genes of the encoding loci are searched to optimize reproductive performance.

Growth hormone (GH) belongs to a family of somatolactogenic hormones which include placental lactogen, prolactin and anthers hematopoietic growth factors (Cosman et al., 1990). GH is an anabolic hormone synthesized and secreted by the somatotroph cells, it is a major regulator of postnatal growth and metabolism in mammals, and it plays an important role in lactation, protein, lipid and carbohydrate metabolism, tissue growth, and fertility in cows (Akers, 2006; Lucy, 2008; Thidar et al., 2008). The growth promoting and metabolic actions of GH are generally mediated by the insulin-like growth factor I (IGF-I) (Ramesha et al., 2015).

GH is a polypeptide hormone with a sequence 191 amino acid, its length is approximately 1800 bp and its production is linked to chromosome region 19q26 in the bovine genome (Hediger et al., 1990) with five exons and four introns.

Several polymorphisms of the bovine GH gene have been described and three polymorphic sites have been identified within the fifth exon of this gene (Yao et al., 1996)

Many researchers have studied the associations of GH gene polymorphisms with production traits in dairy or beef cattle (Balogh et al., 2009a; Heidari et al., 2012) owing to the critical role of GH with respect to growth, body composition, metabolism regulation, lactation and mammary gland development (Lagziel and Soller, 1999). A significant relationship between polymorphisms in the bovine GH gene and lactation performance has been reported (Zhou et al., 2005; Balogh et al., 2009b; Mullen et al., 2010).

Lucy et al. (1993) reported a polymorphic site for *Alu*I restriction endonuclease, due to cytosine to guanine transversion at position 2141 which induced a change of the leucine (L) into a valine (V) in the amino acid sequence at position 127 of the protein chain of bovine GH. The genotype of GH *Alu*I polymorphism was reported to be associated with birth weight in dairy cattle (Biswas et al., 2003) and mature live weight in beef cattle (Zwierzchowski et al., 2001). Current knowledge regarding the interrelation between *Alu*I polymorphism and reproduction is limited, and only a few studies have investigated this association.

No significant association has been reported between *Alu*I polymorphism and the following traits: age at the first calving (Kovács et al., 2006; Grossi et al., 2015), the number of oocytes suitable for in vitro maturation, the number of matured oocytes, the mean oocyte diameter, and the number of embryos produced (Lechniak et al., 2002). However, a significant association was observed for milk yield, milk fat and protein (Dybus, 2002; Kovács et al., 2006), in addition to beef bulls non-return rates (Lechniak et al., 2002).

The GH gene was digested with *Msp*I restriction endonuclease; GH-*Msp*I polymorphism is located in intron 3 of the GH gene at position 1547 (Zhang et al., 1993). As a consequence, two alleles occur, and *Msp*I (-) contains a T insertion at the +837 position and a C–G transition at the +837 position (Lee et al., 1994).

To date, several studies have investigated the association of GH-*Msp*I polymorphism with milk and meat production traits (Zhou et al., 2005; Beauchemin et al., 2006; Katoh et al., 2008), but only a few studies have examined its effect on reproductive traits (Unanian et al., 2002; Lechniak et al., 2002; Gorbani et al., 2009).

Based on the abovementioned evidence, the present investigation was carried out to identify GH-*Alu*I and GH-*Msp*I polymorphisms and uncover their association with reproductive traits in Holsteins dairy cattle.

## 2 Materials and methods

### 2.1 Experimental design

Four hundred and ten (410) Holstein dairy cows from the Agricultural Development farm in northern Tunisia were included in the present work. These animals were either imported as pregnant heifers from European countries or were born on the farm.

Cows were observed for signs of estrus twice daily, as well as during the morning and afternoon milking. Animals detected in estrus were artificially inseminated. Pregnancy diagnostic by rectal palpation was performed during the 60–70 days after insemination, and pedigree information was available for all cows in the herd.

All cow milk production and reproductive performance data were systematically recorded spanning the time period from January 2013 to December 2017. The reproduction data were derived from the national Office of Livestock and Pasture (OEP) database.

### 2.2 Blood samples

Five milliliters of venous blood was collected under sterile conditions, from the jugular vein of animal using a 5 mL polypropylene centrifuge tube containing 0.5 mL of 2.7 % EDTA solution as an anticoagulant. The experiment was carried out with the approval of the Institutional Animal Ethics Committee (IAEC). The centrifuge tube was tightly capped and shaken gently to facilitate the thorough mixing of the blood with the anticoagulant. Blood samples were transported to the laboratory in an icebox containing ice packs and stored in a refrigerator at -20 ${}^{\circ }$C until the isolation of the DNA was carried out.

### 2.3 Genotyping

Genomic DNA was isolated from frozen blood using the NucleoSpin Blood kit (innuPREP Blood DNA Mini Kit) following the manufacturer's instructions. The integrity of the DNA was examined using electrophoresis on a 1 % agarose gel, and acceptable samples were used for PCR reaction.

Genotyping was performed using a PCR reaction mixture containing 4 µL of genomic template, 2.5 µL of buffer, 2.5 µL of dNTP, 2.5 µL of each of the primers (F/R), 0.2 µL of the heat-resistant DNA polymerase (Taq polymerase) and 10.8 µL of autoclaved distilled water in a final volume of 25 µL. The primers used were designed to amplify 428 and 329 bp GH gene fragments. Table 1 shows the primers used for amplification.

PCR was carried out with an initial step of 95 ${}^{\circ }$C for 5 min, followed by 35 cycles of 95 ${}^{\circ }$C for 30 s, 59 ${}^{\circ }$C or 60 ${}^{\circ }$C for 30 s (for *Alu*I and *Msp*I, respectively), 72 ${}^{\circ }$C for 30 s and a final extension of 72 ${}^{\circ }$C for 10 min. The PCR amplification yielded 428 and 329 bp fragments that were revealed using electrophoresis on a 2 % agarose gel.

Restriction endonuclease *Alu*I and *Msp*I were used to digest a 428 and 329 bp PCR product, respectively. Polymerase chain reaction-restriction fragment length polymorphism (PCR-RFLP) reaction requires 12 µL of the PCR product, 0.2 µL of BSA (bovine albumin serum), 2.5 µL of the reaction buffer, 0.3 µL of the restriction enzyme, 10 µL of autoclaved distilled water in a total volume of 25 µL incubated at 40 ${}^{\circ }$C for at least 11 h. The PCR-RFLP fragments were separated using horizontal electrophoresis in 2.5 % agarose gels stained with ethidium bromide and visualized under a UV transilluminator, the gel was documented using Gel Doc equipment, and the genotypes were determined according to band sizes.

Allele and genotype frequencies were calculated using genotyping data from each gene based on the population analyzed. Chi-square statistics (χ2) were used to check whether the populations were consistent with the Hardy–Weinberg equilibrium.

**Table 1:** Primer sequences, sizes of the amplified fragments in PCR and sizes of the observed genotypes.

Locus	Primers (5${}^{\prime }$-3${}^{\prime }$)	Size	Restriction	Digestion product	References
		(bp)	enzyme	size (bp)	
GH-	F 5${}^{\prime }$-CGGACCGTGTCTATGAGAAGCTGAAG-3${}^{\prime }$	428	*Alu*I	LL 265, 96, 51	Trakovická
*Alu*1	R 5${}^{\prime }$-GTTCTTGAGCAGCGCGTCGTCA-3${}^{\prime }$			LV 265, 147, 96, 51	et al. (2013)
				VV 265, 147	
GH-	F (5${}^{\prime }$-CCCACGGGCAAGAATGAGGC-3${}^{\prime }$)	329	*Msp*I	(-/-) 329	Suwiti et al.
*Msp*I	R (5${}^{\prime }$-TGAGGAACTGCAGGGGCCCA-3${}^{\prime }$)			(+/-) 329, 224, 105	(2017)
				(+/+) 224, 105	

### 2.4 Reproductive traits

Lactation and several other reproduction traits were used to investigate the effect of the polymorphisms studied on the performance of dairy cows. All data regarding cow milk production and reproductive performance were systematically recorded spanning the time period from January 2013 to December 2017; the following traits were derived from these records: the integrated age at first calving (AGE_1CALV), the calving interval (CI), the number of inseminations per conception (NINS) and the days open (DO; the interval between calving and the next successful insemination). These traits are believed to be the most important and available parameters of reproductive efficiency for the herd. Descriptive statistics for these traits are presented in Table 2.

**Table 2:** Descriptive statistics for reproductive parameters.

Trait	No. records	Mean	SD	Minimum	Maximum
AGE_1CALV	410	806.044	80.093	657.000	1331.000
NINS	1119	1.869	1.182	1.000	8.000
CI	709	443.668	105.979	243.000	882.000
DO	709	166.931	105.546	34.000	606.000

### 2.5 Statistical analysis

The statistical analysis was performed by repeated measures using the MIXED procedure in SAS software (SAS 9.0 for Windows). The resulting mixed linear model included the following effect which was used to evaluate the impact of *GH* polymorphisms on reproduction traits of cows:

$$\begin{array}{cc} y_{ijklmn} & =\mu +{\mathrm{GAlu}}_{i}+{\mathrm{GMsp}}_{j}+{\mathrm{origin}}_{k}+{\mathrm{season}}_{l}\\ & +{\mathrm{parity}}_{m}+{\mathrm{lameness}}_{n}+e_{ijklmn}, \end{array}$$

where y is the variable dependent on animal, μ is the overall mean, GAlu${}_{i}$ is the fixed effect of the GH-*Alu*I genotype (LL, LV, VV), GMsp${}_{j}$ is the fixed effect of the GH-*Msp*I genotype (-/-, +/-, +/+), origin${}_{k}$ is the fixed effect of the cows' country of origin (k=3), season${}_{l}$ is the fixed effect of the of calving season (l can represent a value from 1 to 4 which refers to winter: December–February, spring: March–May, summer: June–August, autumn: September–November, respectively), parity${}_{m}$ is the fixed effect of the order of parity (m=4), lameness${}_{n}$ is a fixed effect that indicates whether the cow has been diagnosed with lameness or not (n=2) and $e_{ijklmn}$ is the random residual error.

The F statistic and the T statistic were used to compare the effect of each factor. A probability of p≤0.05 and p≤0.01 were considered to be statistically significant and highly significant, respectively.

## 3 Results

### 3.1 Gene frequency

The DNA isolated was of a good quality. The 428 and 329 pb fragments from the GH gene were characterized and successfully amplified from the DNA of each sample; this indicated a strong conservation of the DNA sequence existing in cattle.

The χ2 test showed that the allele frequency and genotypes of GH-*Alu*I and GH-*Msp*I were in Hardy–Weinberg equilibrium.

Visualization under UV light revealed that GH-*Alu*I exhibited three band patterns. Fragments that represent the leucine variant (LL) gave rise to the 265//96//51 pb band, *Alu*I fragments gave rise to the 265//147 pb band that represented the valine variant (VV), and the (LV) pattern gave rise to the 265//147//96//51 pb band.

The following DNA restriction fragments were also obtained as a result of *Msp*I restriction: 329 bp for the *Msp*I(-/-) genotype, 329//224//105 bp for the *Msp*I(+/-) genotype and 224//105 bp for the *Msp*I(+/+) genotype.

Table 3 shows genotypic and allelic frequencies of GH-*Alu*I and GH-*Msp*I polymorphisms in the herd studied. GH-*Alu*I genotype frequencies were 77.75, 18.59 and 3.66 for LL, LV and VV, respectively. These results showed that the L allele was significantly more frequent than the V allele (87.045 vs. 12.955).

The GH-*Msp*I frequency distribution showed that the *Msp*I (+/+) genotype was the most abundantly found (55.50), followed by *Msp*I (+/-) (29.13). The least frequently found genotype was *Msp*I (-/-) (15.37). The *Msp*I (+) allele was more frequent than the *Msp*I (-) allele (70.065 vs. 29.935).

**Table 3:** Genotypic and allelic frequencies (%) in the GH-*Alu*I and GH-*Msp*I polymorphisms.

Gene locus	Polymorphism	Genotype frequency			Allele frequency	
GH	*Alu*I (L/V)	LL	LV	VV	L	V
		77.75	18.59	3.66	87.045	12.955
	*Msp*I (-/+)	(-/-)	(+/-)	(+/+)	(-)	(+)
		15.37	29.13	55.50	29.935	70.065

### 3.2 Factors affecting reproduction traits 

The investigation of the effect of non-genetic factors on reproduction traits showed that parity only had a significant effect on the NINS (p<0.0001) but had no effect on the CI or the DO. The cows' country of origin significantly affected the NINS (p=0.0003) and the AGE_1CALV (p=0.0009) but had no effect on the CI or the DO. The diagnosis of lameness had a highly significant effect on the CI (p=0.0009) and the DO (p=0.0025), a significant effect on the AGE_1CALV (p=0.0211) and no effect on the NINS. The calving season had no impact on any of the traits analyzed.

As shown in Table 4, genetic polymorphisms were found to be related to the abovementioned traits. The GH-*Alu*I significantly affected the NINS (p<0.0001), the CI (p=0.0005) and the DO (p=0.0007) but did not affect the AGE_1CALV. However, the GH-*Msp*I had no effect on any of the traits analyzed.

**Table 4:** The effect of sources of variation on reproductive traits. DDL refers to degrees of freedom, and Pr refers to probability.

Factors	NINS (DDL=402)		CI (DDL=319)		DO (DDL=319)		AGE_1CALV	
							(DDL=399)	
	F	Pr ⩽0.05	F	Pr ⩽0.05	F	Pr ⩽0.05	F	Pr ⩽0.05
Parity	81.36	<.0001	1.27	0.2835	1.35	0.2599		
Origin	8.40	0.0003	0.09	0.9147	0.13	0.8762	7.17	0.0009
Season	2.15	0.0930	1.06	0.3666	0.81	0.4889	0.20	0.8948
Lameness	2.83	0.0935	11.30	0.0009	9.30	0.0025	5.36	0.0211
GH-*Alu*I	18.44	<.0001	7.75	0.0005	7.46	0.0007	0.99	0.3730
GH-*Msp*I	0.96	0.3852	1.72	0.1806	2.08	0.1270	0.37	0.6924

The effect of genetic polymorphisms on reproductive traits was also compared to the GH-*Alu*I and GH-*Msp*I variants (Table 5). The replacement of the L allele by the V allele was found to lead to an increase in the NINS, the CI and the DO, and the performance of the LL genotype was significantly higher compared with similar values of the VV and LV genotypes. Cows with the LL genotype had the lowest NINS (-0.5892) compared with cows with VV and LV genotypes – the difference was highly significant (p<0.0001).

The GH-*Alu*I polymorphism had a substantial effect on the CI (p<0.0001), and the LL genotype shows a shorter CI (-80.76 days) compared with the VV genotype. The difference between the LV and VV genotypes was also considerable (p=0.0021) with a shorter CI for the LV genotype (-69.68 days). The statistical model that estimates the genotype effect revealed that the LL and LV genotypes were significantly associated with the DO (p=0.0002 and p=0.0030, respectively), and that LL and LV cows tend to conceive 78.6 and 66.87 days before VV cows, respectively. There was no effect of the substitution of the L allele by the V allele on the AGE_1CALV: VV cows tended to have their first calving 15.22 and 27.10 days later than LV and LL cows, respectively, but the differences were not statistically significant.

There was no detectable significant effect of the GH-*Msp*I on the traits analyzed, although polymorphism had a suggestive effect on the CI (p=0.0926) and the DO (p=0.0714). *Msp*I (+/-) cows tended to have the longest CI and DO, but the difference was still not significant.

**Table 5:** Association of GH-*Alu*I and GH-*Msp*I genotypes with reproduction trait. DDL refers to degrees of freedom, and Pr refers to probability.

Traits	Genotypes		Estimate	Standard error	t value	Pr ⩽0.05
NINS	Intercept	2.7969	0.1844	15.17	<.0001	
(DDL=402)	GH-*Alu*I	LL	-0.5892	0.1296	-4.55	<.0001
		LV	-0.313	0.1391	-2.25	0.0248
		VV	0	--	--	--
	GH-*Msp*I	+ +	0.09197	0.06873	1.34	0.1816
	+-	0.08968	0.07500	1.20	0.2325	
		--	0	--	--	--
CI	Intercept	483.72	26.3921	18.33	<.0001	
(DDL =319)	GH-*Alu*I	LL	-80.7633	20.9099	-3.86	0.0001
		LV	-69.6775	22.4212	-3.11	0.0021
		VV	0	--	--	--
	GH-*Msp*I	+ +	7.7423	11.5392	0.67	0.5027
		+-	21.1894	21.1894	1.69	0.0926
		--	0	--	--	--
DO	Intercept	207.87	26.4252	7.87	<.0001	
(DDL=319)	GH-*Alu*I	LL	-78.6020	20.8400	-3.77	0.0002
		LV	-66.8659	22.3395	-2.99	0.0030
		VV	0	--	--	--
GH-*Msp*I	++	7.3830	11.4916	0.64	0.5210	
		+-	22.6165	12.5016	1.81	0.0714
		--	0	--	--	--
AGE_1CALV	Intercept	836.21	24.7533	33.78	<.0001	
(DDL =399)	GH-*Alu*I	LL	-15.2213	21.2815	-0.72	0.4749
		LV	-27.0951	22.8912	-1.18	0.2373
		VV	0	--	--	--
	GH-*Msp*I	+ +	-5.6450	11.0821	-0.51	0.6108
		+-	1.6283	12.1276	0.13	0.8933
		--	0	--	--	--

A statistical evaluation of the association of the GH-*Alu*I–*Msp*I combined genotypes on reproductive traits showed a significant effect on the NINS (p<0.0001), the CI (p=0.0024) and the DO (p=0.0022), although no effect was found on the AGE_1CALV (Table 6).

The estimate of the VV/- genotype intercept for the NINS, the CI, the DO and the AGE_1CALV is 2.610, 498.45, 218.79 and 812.61, respectively. However, differences in the combined genotype performance is only significant for the CI and the DO.

**Table 6:** The effect of combined genotypes on reproductive traits in dairy cows.

GH-*Alu*I–*Msp*I	NINS		CI		DO		AGE_1CALV	
Pr >F	<.0001		0.0024		0.0022		0.7168	
Genotypes	Estimation	Pr |>t|	Estimation	Pr >|t|	Estimation	Pr >|t|	Estimation	Pr >|t|
Intercept	2.6102	<.0001	498.45	<.0001	218.79	<.0001	812.61	<.0001
LL ++	-0.3243	0.2134	-85.8642	0.0323	-80.6255	0.0428	-0.8995	0.9842
LL +-	-0.3230	0.2184	-77.730	0.0544	-70.0946	0.0805	9.6545	0.8320
LL --	-0.3700	0.1659	-95.754	0.0210	-89.4670	0.0298	13.9488	0.7624
LV ++	0.01667	0.9504	-77.2337	0.0615	-69.9055	0.0881	-1.5947	0.9727
LV +-	-0.09708	0.7281	-59.7961	0.1666	-54.1317	0.2071	-6.3468	0.8953
LV --	-0.2377	0.4164	-88.3185	0.0495	-82.0046	0.0662	-29.8283	0.5560
VV ++	0.1470	0.6435	-62.1095	0.2281	-58.7710	0.2511	28.1525	0.5988
VV +-	0.6081	0.0769	50.7791	0.3273	58.9377	0.2537	19.4899	0.7434
VV --	0	–	0	–	0	–	0	–

## 4 Discussions

There were no genetic factors (namely parity, origin, calving season and lameness) that clearly accounted for the reproduction traits examined in this study.

Parity showed a significant relationship with the NINS, with primiparous cows requiring a lower number of inseminations per conception than pluriparous cows. The impact of the cow's origin with respect to the NINS and the AGE_1CALV provided evidence that exported cows had better reproductive performance than native cows in this study. Regarding the antagonist correlation between lameness and reproduction traits, cows that were diagnosed with lameness did not perform as well as cows that were not diagnosed. In addition, differences in the feeding and climatic features of the calving season had no effect on the traits analyzed except for the fact that the NINS was slightly influenced by calving season.

The majority of cows were homozygous for the leucine allele (L; 77.75 %); only 3.66 were homozygous for the valine allele (V), whilst 18.59 % were heterozygous. Therefore the frequency of the V allele was lower than the L allele (87.045 vs. 12.955). Allele and genotype frequencies for the GH-*Alu*I polymorphisms given in this work were in agreement with those reported in previous studies, which have been discussed extensively in Holstein dairy cows (Balogh et al., 2009b; Ruprechte et al., 2011; Grossi et al., 2015).

However, other findings have shown the dominance of the heterozygous genotype LV and the absence of the homozygous genotype VV (Aruna et al., 2004; Hadi et al., 2015).

The most frequent genotypes for GH-*Msp*I were homozygous *Msp*I(+/+) (55.50), whereas the homozygous *Msp*I(-/-) genotype was the least frequent (15.37). Hence, the dominance of the *Msp*(+) allele was evident compared with the *Msp*I(-) allele (70 065 vs. 29 935). Results of the present work are in agreement with those published by Dybus et al. (2002) in Polish Black-and-White cattle and by Zhou et al. (2006) in China Holstein cows. However, Hartatik et al. (2018) reported a higher frequency of the *Msp*I(+/-) genotype than the *Msp*I(-/-) and *Msp*I(+/+) genotypes in Pesisir cattle and cross breeds, in addition to the fact that *Msp*I(-) allele was more frequent than the *Msp*I(+) allele (0.533 vs. 0.467).

There are only a few studies that have investigated the relationship between GH-*Alu*I and GH-*Msp*I polymorphisms and reproductive traits in Holstein dairy cattle. Evaluations of this relationship in this work showed a significant effect of the GH-*Alu*I on the NINS, the CI and the DO but no effect on the AGE_1CALV. Furthermore, no significant association between the GH genotype and the interval from calving to first ovulation was observed by Balogh et al. (2009b).

Similarly, no obvious association of GH with AGE_1CALV and CI was observed in Canchim and Hungarian Holstein–Friesian cattle (Kovacs et al., 2006; Grossi et al., 2015) and with AGE_1CALV in a Hungarian Holstein–Friesian bull dam population (Katalin et al., 2006). Moreover, Kovacs et al. (2006) reported no noteworthy effect of GH-*Alu*I on the CI, the number of calves or the dry period in a Hungarian Holstein–Friesian bull dam. Ruprechter et al. (2011) found no effect of the GH genotype on the calving–first service interval, the number of services per conception or the total pregnancy rate, and no other significant effects were observed with respect to the number of matured oocytes or the number of embryos produced (Lechniak et al., 2002).

In our study, the LL genotype is considered the most favorable with respect to reproductive traits, as cows carrying the LL genotype showed better performance than LV and VV cows – this difference was highly significant for NINS, the CI and the DO. Lechniak et al. (1999) reported no significant genotype effect for lower ejaculate volumes for leucine homozygous bulls and greater non-return rates of VV beef bulls at 60 days postpartum.

Sabour et al. (l997) reported that V/V genotypes were related to better milk traits, especially higher milk protein compared with the other genotypes. These findings are in line with our result if we consider the established antagonistic correlation between reproduction and the milk production function, which means that the GH genotype may directly or indirectly affect reproduction performance. In addition, numerous previous studies have suggested an additive effect of the L allele on milk production and contents with LL cows reportedly yielding more milk, fat and protein than LV and VV cows (Lucy et al., 1991; Shariflou et al., 2000; Dybus, 2002).

This examination of the impact of the GH locus on reproductive traits showed that GH-*Msp*I polymorphism only had a suggestive, non-significant effect on the CI and the DO. To date, few studies have investigated the effect of GH-*Msp*I polymorphism on reproductive traits of bulls, and the results of these studies have reported a significant association between GH-*Msp*I polymorphisms and the following sperm quality traits in bulls: fresh sperm motility, sperm concentration, minor and major defects, scrotal circumference and testicular growth after puberty (Lechniak et al., 2002; Unanian et al., 2002; Gorbani et al., 2009); the reproductive traits of beef cattle have also been studied (Anggraeni et al., 2017).

Arango et al. (2014) reported that *Msp*I polymorphism is associated with age at first service, first birth, first postpartum service and second birth. Furthermore, the *Msp*I (+/+) genotype had the greatest effect on the age at first service and was considered the optimal genotype for reducing age at first service, first birth, first postpartum service and second birth. In addition, several inconsistent results have reported the effect of GH-*Msp*I polymorphism on growth and milk production traits (Dybus et al., 2004; Pereira et al., 2005; Zhou et al., 2006; Katoh et al., 2008). *Msp*I(+/+) cows are considered to yielded more milk, fat and protein than +/- individuals (Zhou et al., 2006).

Significant differences between cows with different GH-*Alu*I–*Msp*I combined genotypes were found at the 5 % level for the CI; cows carrying the LL/- genotype showed significantly (p=0.021) better performance, and their CI was 95.754 days lower than VV/- cows. The GH-*Msp*I–*Alu*I combined genotypes also significantly affected the NINS (P<0.0001); cows with the VV/+- genotype needed the highest NINS in the herd, although this tendency was not significant.

The effect of the combined genotypes was also notable with respect to the DO (p=0.0022), further confirming the difference between cows of different genotypes. The LL/- cows were significantly (p=0.0298) associated with the shortest DO compared to the estimated intercept of the VV/- genotype. However, no significant association was found between cows with different GH-*Alu*I–*Msp*I combined genotypes and the AGE_1CALV.

As previously stated, the *Alu*/LL genotype showed the best relationship with reproductive traits, whereas the GH-*Msp*I only had a suggestive effect on the CI and the DO. Hence, the LL/- combined genotype can be expected to be associated with better performance. Very few studies have investigated the effect of GH-*Alu*I–*Msp*I combined genotypes in dairy cattle. However Dybuset al. (2004) reported that VV/++ cows produced milk with the highest fat and protein content.

## 5 Conclusions

In this study, *Alu*I and *Msp*I polymorphisms of the bovine GH gene were identified in a population of Holstein dairy cows in Tunisia which displayed L allele and *Msp*I(+) allele dominance. GH-*Alu*I significantly affected the NINS, the CI and the DO; however GH-*Msp*I had no effect on any of the traits evaluated except for a slight tendency on the CI and the DO. GH-*Msp*I–*Alu*I combined genotypes had a highly significant affect on the NINS, the CI and the DO, and cows with the LL/- genotype tended to have better reproductive traits.

Under field conditions, the detection of polymorphisms in genes related to reproductive traits and the identification of the allele which results in a phenotype of interest can be considered as candidate markers of reproductive traits for cattle breeders.

## Data Availability

The data are not publicly accessible; they are the property of the National Livestock and Pasturing Office (OEP) of Tunisia.
